# Low temperature solution process-based defect-induced orange-red light emitting diode

**DOI:** 10.1038/srep17961

**Published:** 2015-12-09

**Authors:** Pranab Biswas, Sung-Doo Baek, Sang Hoon Lee, Ji-Hyeon Park, Su Jeong Lee, Tae Il Lee, Jae-Min Myoung

**Affiliations:** 1 Department of Materials Science and Engineering, Yonsei University, 50 Yonsei-ro, Seodaemun-gu, Seoul 120–749, Republic of Korea; 2 College of BioNano Technology, Gachon University, 1342 Seongnamdae-ro, Sujeong-gu, Seongnam-si, Gyeonggi-do 461–701, Republic of Korea

## Abstract

We report low-temperature solution-processed p-CuO nanorods (NRs)/n-ZnO NRs heterojunction light emitting diode (LED), exploiting the native point defects of ZnO NRs. ZnO NRs were synthesized at 90 °C by using hydrothermal method while CuO NRs were synthesized at 100 °C by using microwave reaction system. The electrical properties of newly synthesized CuO NRs revealed a promising p-type nature with a hole concentration of 9.64 × 10^18^ cm^−3^. The current-voltage characteristic of the heterojunction showed a significantly high rectification ratio of 10^5^ at 4 V with a stable current flow. A broad orange-red emission was obtained from the forward biased LED with a major peak at 610 nm which was attributed to the electron transition from interstitial zinc to interstitial oxygen point defects in ZnO. A minor shoulder peak was also observed at 710 nm, corresponding to red emission which was ascribed to the transition from conduction band of ZnO to oxygen vacancies in ZnO lattice. This study demonstrates a significant progress toward oxide materials based, defect-induced light emitting device with low-cost, low-temperature methods.

Solid-state light emitting diode (LED) effectively rules out the limitations of conventional lamps with its small and compact size, better performance, and less power consumption capacity. It can also produce light with different colors making it more useful for applications in display technology. After achieving white LED, cheap and stable light emitting devices have been remained the core area of research. As a matter of consequence, there are continuous efforts in scientific community to apply the materials which are stable, abundant, cheap, non-toxic, and easy to process. The LEDs with conventional III–V semiconductors (typically, GaN and GaAs based materials) are expensive because of their high-end vacuum growth techniques (metal organic chemical vapor deposition, molecular beam epitaxy, and other vapor deposition techniques) with high temperature in order to maintain high purity. In contrast, oxide semiconductors are easily available, cheap, easy to process, non-toxic, and chemically stable in harsh environment. Zinc oxide (ZnO) is such a promising functional material which can be efficiently used in optoelectronic devices. It has a direct band gap of 3.3 eV with a remarkably large exciton binding energy of 60 meV (at 300 K) which is 2.5 times to that of its rival, GaN^1^,^2^. This high exciton binding energy facilitates it to use at higher temperature (>300 K). On the other hand, ZnO has a diverse range of nanostructures such as nanorods (NRs), nanotubes, nanowires, nanoflowers, and nanobelts^3^,^4^,^5^,^6^,^7^. These nanostructures offer an advantage for light emission due to increased junction area and improved carrier confinement capacity^8^,^9^. In particular, vertically grown ZnO NRs can act as natural waveguide cavities which help the emitted light to travel to the top of the device^10^. This unidirectional emission of light also in turn enhances the intensity. Most significantly, ZnO NRs have a large number of intrinsic defect states which can also be used as the emission sources in visible LEDs^11^,^12^.

Though it has all the above advantages and has outstanding potential for LED application, as-grown ZnO always shows n-type conductivity. Repeated attempts have been remained unsuccessful to achieve stable and reproducible p-type conductivity with reasonably high carrier concentration, jeopardizing its effective applications in homojunction LEDs^13^,^14^. This has instigated ZnO to fabricate p-n heterojunction LEDs using other suitable p-type material^15^. It was found that copper oxide (CuO) is such an as-grown p-type material which can be grown using low-temperature solution process^16^. At the same time, the material is cheap, chemically stable, and non-hazardous, satisfying the present day demand. Most importantly, CuO is a well-established hole transport material which is indispensable for LEDs^17^. Also, the material has a direct bandgap of 1.2 to 1.5 eV making it suitable to apply in optoelectronic devices^18^.

Thus, we fabricated p-CuO NRs/n-ZnO NRs heterojunction diode and demonstrated its performance as LED. A comprehensive study using photoluminescence was carried out to investigate the probable emission sources. Keeping in view at the world-wide demand for cheap LED, low-temperature solution processes were applied to synthesize ZnO and CuO NRs. Here, CuO NRs were grown using a microwave-assisted novel method. These results show that native point defects in ZnO NRs can effectively be used in LEDs to achieve visible range emission.

## Results

**Figure 1:**
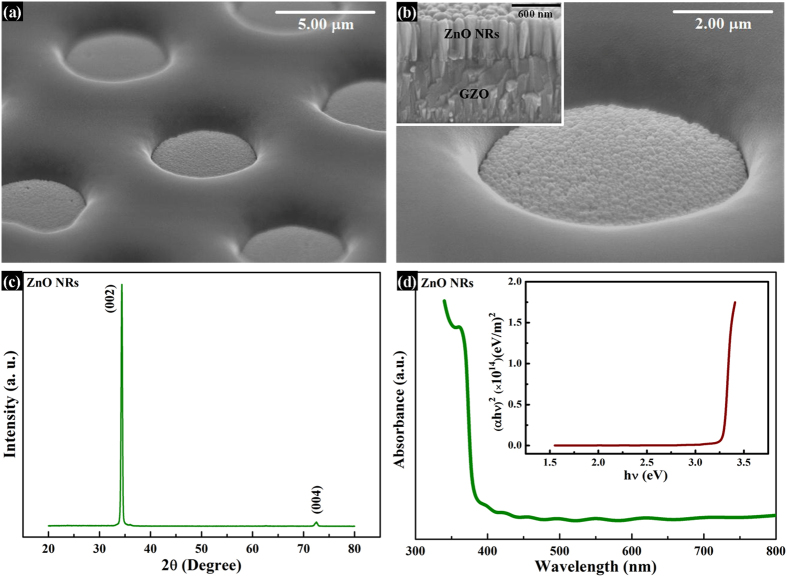
Surface morphology, structural and optical characteristics of ZnO NRs. Tilted view of SEM images showing ZnO NRs grown on GZO coated glass in (**a**) assembly of PR pattern, and (**b**) single PR pattern. The inset of fig. (**b**) is the cross-sectional view of the grown ZnO NRs in the PR pattern indicating a length of 600 nm; (**c**) XRD pattern of single crystalline, c-axis oriented ZnO NRs; (d) The UV-Vis absorption spectra of the NRs. Inset of (**d**): The corresponding Tauc plot indicating a direct optical bandgap of 3.3 eV.

Figure 1(a,b) show the SEM images of grown ZnO NRs on the patterned GZO-coated glass substrate. It is observed that the ZnO NRs are closed-packed, uniform, and vertically aligned inside the photoresist (PR) pattern. The cross-sectional view of the NRs in the inset of the Fig. 1(b) indicates a thickness of around 600 nm after 1 h of hydrothermal growth. The x-ray diffraction (XRD) pattern of the NRs is shown in Fig. 1(c). The sharp peak at 2θ value of 34.38 degree originates from (002) plane of hexagonal wurtzite lattice of ZnO. The small peak at 2θ value of 72.8 degree originates from (004) plane of the same^19^. This result signifies that the ZnO NRs are single crystalline and highly oriented along c-axis direction. Figure 1(d) shows the UV-VIS absorption spectrum with a sharp absorption at 376 nm. The optical bandgap was calculated to be 3.3 eV by using Tauc plot which is shown in the inset of Fig. 1(d)^20^,^21^.

**Figure 2:**
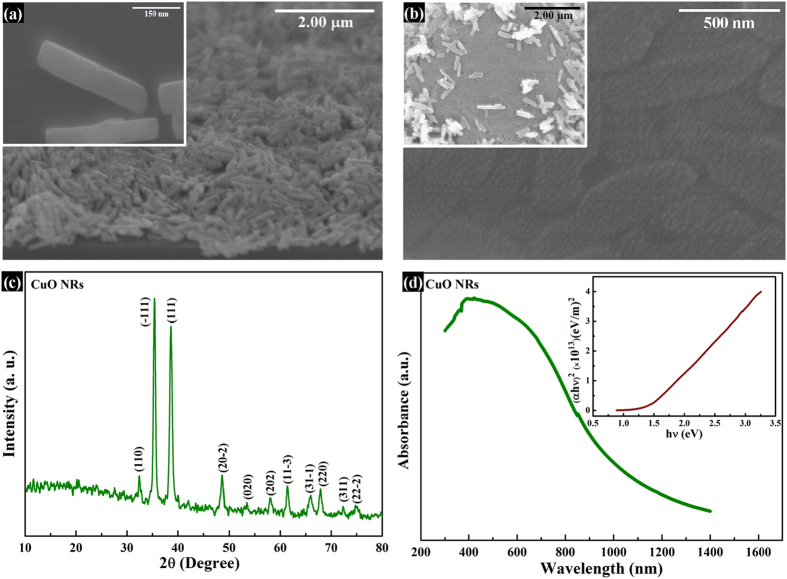
Surface morphology, structural and optical characteristics of CuO NRs. The SEM images of CuO NRs after (**a**) PDMS transfer on a bare and unpatterned substrate, and after (**b**) PDMS transferring, rubbing, and pressing in a 5 μm pattern. The inset of (**a**) is the corresponding magnified view of the image indicating an average length and breadth of 400 nm and 50 nm of the NR, respectively. The inset of (**b**) shows the full view of a pattern with uniform, compact, and smooth CuO layer after rubbing and pressing; (**c**) XRD pattern of CuO NRs, predominantly in the direction of (111); (**d**) The UV-Vis absorption spectra of CuO NRs, of which the Tauc plot is also shown in the inset indicating an optical bandgap of 1.4 eV.

**Figure 3:**
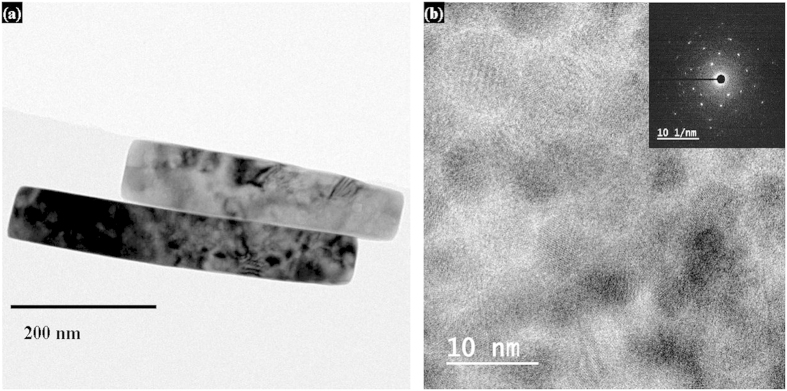
Crystalline property of CuO NRs using TEM. (**a,b**) The TEM images showing the size, shape, and multi-directional lattice fringe of CuO NRs, of which the corresponding SAED pattern is shown in the inset of (**b**).

On the other hand, Fig. 2(a) shows the SEM images of spin-coated CuO NRs on bare substrate. The inset of the figure shows the magnified view. It was observed that the NRs have an average length and width of around 400–500 nm and 50–100 nm, respectively. Most significantly, the novel CuO NRs showed a unique tendency of agglomeration. Figure 2(b) shows CuO NRs layer in the 5 μm circular pattern after transferring, rubbing, and pressing by using polydimethylsiloxane (PDMS). The image confirms that the CuO NRs layer is uniform, smooth, and compact which are essential for electrode deposition. The above-said properties of the CuO NRs help them to create a compact and smooth layer upon rubbing and pressing. The inset of the Fig. 2(b) shows the same with the view of a full pattern. Figure 2(c,d) show the XRD pattern and UV-VIS absorption spectrum of CuO NRs, respectively. It was found that all the peaks were related to standard monoclinic CuO (JCPDS file no. 48–1548 C2/c). The XRD results also indicated that the NRs are purely CuO without any other impurity^22^. The NRs were found to be oriented predominantly along (111) direction. The UV-VIS spectrum and corresponding Tauc plot (inset: Fig. 2(d)) revealed a direct optical bandgap of 1.4 eV which was found to be in accordance with the existing report^23^,^24^. The chemical composition of CuO NRs was investigated by using XPS results which have been added as supplementary information, S1. Figure S1(a) shows the full scan spectra of the material which confirms its purity with no other peak than those related to Cu and O. The narrow-scan Cu 2p spectra in Fig. S1(b) show the peaks related to copper (II) oxide, confirming the nonexistence of copper (I) oxide or any other composition related to copper in the material^25^. Thus, the XPS results revealed the purity of material with its atomic composition. Figure 3(a) shows the bright-field TEM image of CuO, indicating a rod like shape which is in accordance with the SEM images of Fig. 2(a). Figure 3(b) shows the high resolution lattice image confirming polycrystalline nature of the NRs with patches of fringe in various directions. The inset of the Fig. 3(b) shows the selected area electron diffraction (SAED) pattern in reciprocal lattice system denying any long-range regular atomic structure. Room temperature Hall measurements of CuO NRs revealed a remarkably high carrier concentration of 9.64 × 10^18^ cm^−3^ with p-type nature and reasonably good hole mobility of 1.77 cm^2^/V•s with a resistivity of 2.7 Ω•cm. These electrical properties indicate that this facile low-temperature method can be adopted to synthesize CuO NRs for its efficient use as a hole transport layer in LEDs.

**Figure 4:**
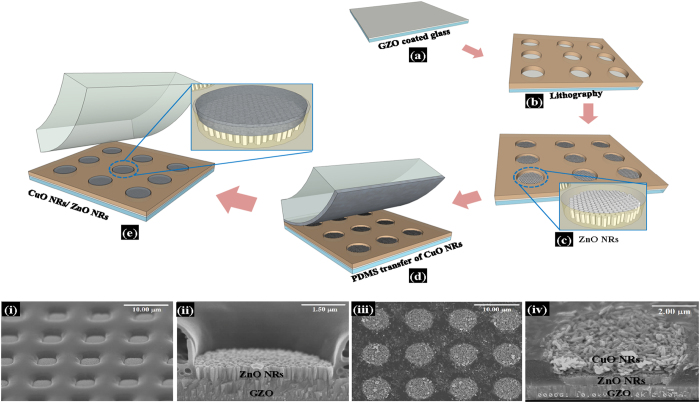
Fabrication of p-CuO NRs/n-ZnO NRs heterojunction. The schematic of the steps (**a–e**) accomplished to fabricate the p-CuO NRs/n-ZnO NRs heterojunction with the SEM images (i–iv) after each major step.

**Figure 5:**
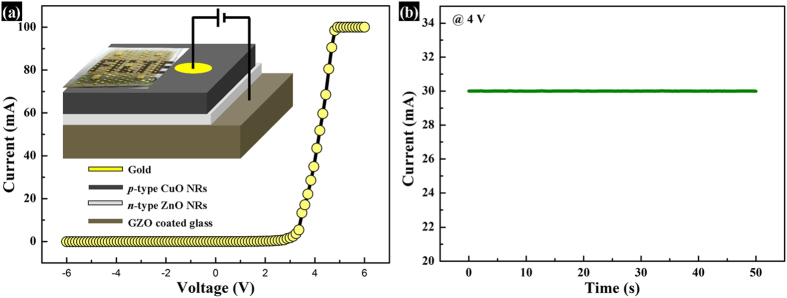
Current-voltage characteristics of the heterojunction diode. (**a**) Current-voltage characteristic of the p-n heterojunction. The inset shows the schematic of the device under dc forward bias. (**b**) The current-flow stability and the endurance characteristic of the device at 4 V for 50 s.

**Figure 6:**
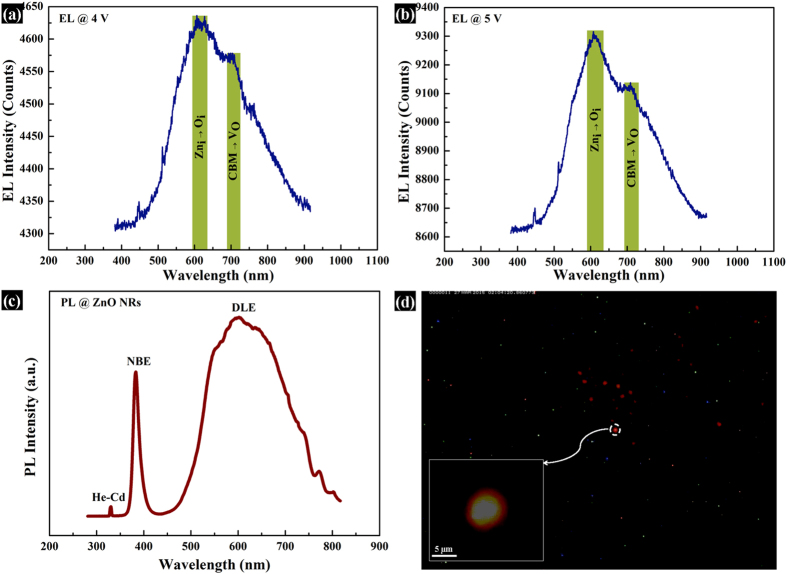
Emission properties of the LED using EL and PL spectra. Electroluminescence (EL) characteristics of the hetrrojunction LED at (**a**) 4 V and (**b**) 5 V, showing major peak at 610 nm corresponding to orange-red light; (**c**) Photoluminescence (PL) of ZnO NRs with a sharp near-band edge emission (NBE) and a broad visible-range defect level emission (DLE); (**d**) The emission image of the LED showing an orange-red color.

The fabrication steps of LED have been schematically depicted in Fig. 4(a–e), whereas, Fig. 4(i–iv) show the corresponding SEM images of the major steps of fabrication. Figure 4(iiii) show the SEM images after deposition of ZnO NRs and CuO NRs, respectively while Fig. 4(ii,iv) show the cross-sectional view of the same, respectively. It can be observed that the thickness of ZnO is 600 nm, whereas that of CuO is around 1 μm. In order to perform dc electrical measurements of the heterojunction, Au electrode was deposited onto CuO using e-beam evaporator. The forward-biased current-voltage (I–V) characteristic, as shown in Fig. 5(a), establishes typical p-n heterojunction diode-nature of the device with a cut-in voltage of 3.2 V and a remarkably high rectification ratio of 10^5^ at 4 V. It was found that in-rush current is a very common phenomenon in LEDs which might lead to permanent damage to the device^26^. Avalanche Joule heating due to radiative recombination of the charge carriers which emit photons along with large number of non-radiative charge carriers can easily damage an LED. Therefore, to protect the diode from uncontrollable in-rush current and subsequent damage, a current compliance of 100 mA was imposed during I–V measurements. The dc forward-biased diode with electrical contacts is schematically depicted in the inset of Fig. 5(a). The diode performed a stable current flow of 30 mA at a voltage stress of 4 V for 50 s, as shown in Fig. 5(b). The high value of rectification ratio and the stability test confirm the superiority of the diode. The electroluminescence behavior of the diode was performed under the dc forward bias of 4 V and 5 V, as shown in Fig. 6(a,b), respectively. The EL intensity has become more than double with an increase in dc forward bias by 1 V, confirming a substantial photon emission with increasing current flow. The emitted photons have a broad range of visible wavelengths corresponding to orange-red light with an eminent peak around 610–620 nm and a small sub-peak around 710–720 nm. To investigate the source of emission, the defect states of ZnO NRs were verified by using room temperature PL, which is shown in Fig. 6(c). Apart from the peaks related to He-Cd laser at 325 nm and near-band-edge (NBE) emission at 380 nm, there is a broad visible-range peak starting from 500 nm upto 800 nm which can be ascribed to deep-level-emission (DLE). The DLE can be attributed to the transitions between the native point defects such as interstitial zinc (Zn_i_), interstitial oxygen (O_i_), zinc vacancy (V_Zn_), and oxygen vacancy (V_O_) which are common in ZnO and most-widely observed in ZnO nanostructures^8^,^9^,^10^,^11^,^12^. The DLE maintains the highest intensity at around 610 nm, corresponding to orange-red light with a minor peak around 720 nm, corresponding to red light. Therefore, the PL spectra evidently disclose that EL emission of orange-red light from ZnO NRs is highly anticipated. Existing literature reported that these defects in ZnO can effectively be used for violet, blue, green, yellow, orange-red, and red emission and thus, combination of the RGB (red-green-blue) colors could lead to low-cost white LED^27^. Figure 6(d) shows the image of the emitted orange-red light from the heterojunction diode under a forward bias of 4 V. The inset of the figure represents the emission from single 5 μm patterned device. Figure S1 (Supplementary Information) shows the same image in blue background, whereas, Video V1 (Supplementary Information) reveals the continuous emission containing those 18 snaps with a time delay of 1 s.

## Discussion

**Figure 7:**
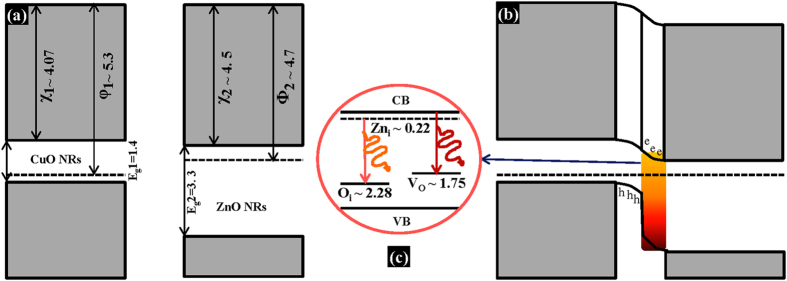
Defect-induced emission mechanism. The schematic band-diagram (**a**) before, and (**b**) after making contact between p-CuO NRs and n-ZnO NRs; (**c**) The emission source of orange-red light depicting the probable charge transitions between the native defects of ZnO NRs. All the values in the diagram are in eV unit.

The possible emission sources have been demonstrated with the help of the schematic band-diagrams, as shown in Fig. 7(a–c). Figure 7(a,b) show the band-diagrams before and after making contact between p-type CuO and n-type ZnO, respectively. The values of electron affinity and work function have been taken from the existing literature to calculate the corresponding band-bending^28^,^29^. The bandgaps of the materials have been considered as the optical bandgaps calculated by Tauc relation (Figs 1(d) and 2(d)). After making contact, the conduction band offset (∆E_C_) and valence band offset (∆E_V_) were calculated to be 1 eV and 2.9 eV, respectively, causing an absolute barrier to carrier flow through the depletion region. We believe that these high values of band offsets obstruct direct flow of charge carriers and facilitate radiative recombination inside the depletion region of the heterojunction. Since the bandgap of CuO is only 1.4 eV, any kind of radiative recombination corresponding to visible-range was ruled out from the CuO side. We assumed that radiative recombination could not happen on CuO side because of its low-crystalline. The visible-range emissions from LED also eliminate the probability of band-to-band transition of ZnO as it would be in ultraviolet range. On the other hand, the ZnO NRs showed a high-intensity broad-range DLE in PL spectrum, confirming the presence of native point defects in undoped ZnO lattice. After verifying the possibilities of all the defect related transitions, we assumed that the eminent peak related to orange-red (610 nm) emission was due to the transition from the defects, Zn_i_ to O_i_. The minor shoulder peak (710 nm) related to red light can be attributed to transitions from conduction band (CB) to V_O_. Therefore, the transitions were believed to be defect-induced and inside the ZnO lattice of the depletion region. The schematic diagram in Fig. 7(c) represents the corresponding transitions and photon emissions. The locations of defect levels inside the bandgap of ZnO were considered according to existing literature^30^,^31^,^32^,^33^. Thus, the ZnO NRs contain large number of Zn_i_, O_i_, and V_O_ defects, as per the investigation from PL and EL. During low temperature (90 °C) synthesis of ZnO NRs, those surface defects were incorporated resulting in an intense DLE in the orange-red region.

## Summary

We have successfully fabricated low-temperature solution-processed oxide materials-based p-CuO NRs/n-ZnO NRs heterojunction LED. CuO NRs were grown using a microwave-assisted novel synthesis method at a temperature of 100 °C. The material claimed a promising electrical property with a high hole concentration of 9.64 × 10^18^ cm^−3^ and showed unique structural nature of agglomeration. The diode showed a remarkably high rectification ratio 10^5^ at 4 V with good stability. Orange-red light emission from the device was observed in the naked eye. The PL spectra of ZnO NRs revealed that it contains a large number of defect states, resulting in a broad and intense deep level emission. The EL spectra confirmed visible range orange-red emission with an eminent peak at 610 nm along with a minor shoulder peak at 710 nm. The lofty peak at 610 nm is due to the transition from Zn_i_ to O_i_, whereas, the latter one at 710 nm is related to the transition from CB to V_O_. Thus, we have achieved less commonly observed orange-red light, exploiting the intrinsic point defects of ZnO NRs, satisfying a step toward low-cost, oxide material based LED.

## Methods

### Materials syntheses and characterization

Prior to grow ZnO NRs, 3 wt% Ga-doped ZnO (GZO) was deposited on glass and GZO-coated glass was used as the substrate. The substrates were cleaned by subsequent sonication in acetone, methanol, and de-ionized (DI) water (18.2 MΩ). Here, we have transferred a circle pattern of 5 μm diameter on the substrates by using optical lithographic technique to achieve continuous, compact, and uniform ZnO NRs. Undoped ZnO NRs were grown onto the patterned GZO-coated substrate in 50 ml of aqueous solution containing 297.49 mg of zinc nitrate hexahydrate (Zn(NO_3_)_2_.6H_2_O) and 140.19 mg of hexamethylenetetramine (HMT) at a temperature of 90 °C for 1 h^34^,^35^,^36^. Simultaneously, CuO NRs were synthesized using a facile low-temperature solution method in CEM’s MARS 6 microwave reaction system. Here, 181.2 mg of copper nitrate trihydrate (Cu(NO_3_)_2_.3H_2_O) and 168.33 mg of potassium hydroxide (KOH) were dissolved in 15 ml of DI water. Then, 2 ml of ethylene glycol was added and the total volume was brought up to 30 ml with nanopure water. The resulting solution was poured in the microwaveable teflon vessel. The vessel temperature was fixed at 100 °C and the reaction was carried out for 2 h. After the completion of reaction, the resulting solution was centrifuged and washed in DI water and ethanol, consecutively. The final solution was dried at 60 °C to get the CuO NRs powder. The surface morphology of the materials was characterized by using Hitachi S5000 scanning electron microscopy (SEM). The structural property of CuO NRs was also characterized by using JEOL’s JEM-ARM 200F transmission electron microscopy (TEM). Rigaku UltimaIV X-ray diffractometer was used to examine the crystallinity of the materials. The optical properties of the materials were investigated using JASCO V-670 UV-VIS spectrophotometer was used. To know the chemical composition and the purity of newly grown CuO NRs, X-ray photoelectron spectroscopy (XPS) was carried out using AXIS–NOVA (KRATOS Inc.) with a monochromatic Al-Ka (1486.6 eV) X-ray source. The electrical properties of CuO NRs were investigated by room temperature Hall measurements in van der Pauw configuration.

### LED fabrication and characterization

The above-said CuO NR powder was deposited on the patterned ZnO NRs by using standard PDMS-transfer-and-rubbing technique. This technique facilitates to fill the 5 μm circular pattern more efficiently unlike spray- or spin-coating technique. By using PDMS, first of all, a monolayer was transferred on the patterned ZnO samples. After transfer, PDMS rubbing technique helps to fill the rest part of 5 μm circular pattern by CuO NRs above ZnO NRs and to create a proper junction. This rubbing technique also assists to make a horizontally aligned uniform layer of CuO NRs on vertically aligned ZnO NRs. This technique resists the formation of continuous layer of CuO on the whole samples and thus, helps to form many cells of p-n heterojunction with a diameter of 5 μm circular pattern. The final pressing by PDMS provide the compactness and smooth surface of the CuO NRs. This helps to deposit Au electrode without any pinholes. The compact and smooth CuO layer in 5 μm circular pattern is shown in Fig. 2(b) with two different resolutions. The fabrication steps have been depicted schematically in Fig. 4. Finally, the devices were annealed at 200 °C for 1 h in Ar ambiance with a flow rate of 1.5 LPM. Using e-beam evaporator Au was deposited on CuO films as the p-type electrode, whereas GZO was considered as the bottom electrode. The electrical measurements of the p-n heterojunction were carried out using semiconductor device analyzer by Agilent Technologies B1500A. The electroluminescence (EL) of the device was investigated by using Andor SOLIS simulation software coupled with a CCD camera (CamWare 64). To investigate the light emission sources from the device, room temperature photoluminescence (PL) measurement was carried out using IK3252R-E He-Cd (325 nm) laser source coupled with MonoRa 320i monochromator and Andor SOLIS simulation package.

## Additional Information

**How to cite this article**: Biswas, P. *et al.* Low temperature solution process-based defect-induced orange-red light emitting diode. *Sci. Rep.* **5**, 17961; doi: 10.1038/srep17961 (2015).

## Supplementary Material

Supplementary Information

Supplementary Video 1
