# Supplementary material for: Low temperature solution process-based defect-induced orange-red light emitting diode
Source: Sci Rep. 2015 Dec 9;5:17961. doi: 10.1038/srep17961 (PMC4673691; doi:10.1038/srep17961)
Supplement: Supplementary Information [file srep17961-s1.pdf]

Supplementary Information

Low temperature solution process-based defect-induced orange-red light emitting diode

Pranab Biswas[1], Sung-Doo Baek[1], Sang Hoon Lee[1], Ji-Hyeon Park[1], Su Jeong Lee[1], Tae Il Lee[2], Jae-Min Myoung[1]

[1]Department of Materials Science and Engineering, Yonsei University, 50 Yonsei-ro, Seodaemun-gu, Seoul 120-749, Republic of Korea.

[2]College of BioNano Technology, Gachon University, 1342 Seongnamdae-ro, Sujeong-gu, Seongnam-si, Gyeonggi-do 461-701, Republic of Korea.

*E-mail address: jmmyoung@yonsei.ac.kr

Figures:

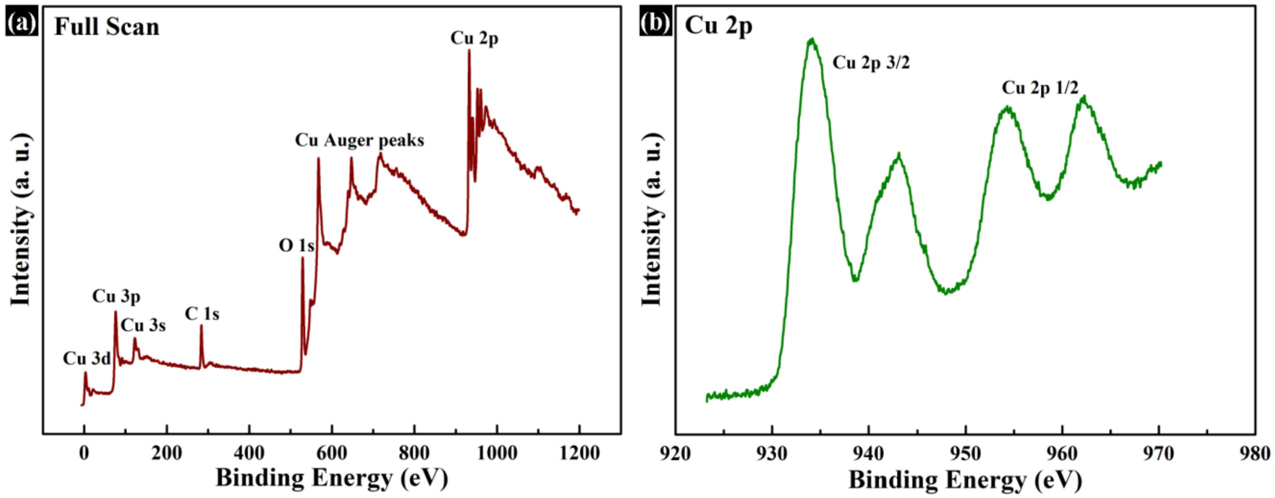

Figure S1 | Chemical composition of novel CuO NRs. (a) Full scan XPS spectra of CuO NRs showing the purity of the material with the peaks related to copper and oxygen. (b) Cu 2p narrow scan spectra of CuO NRs showing the peaks corresponding to Cu 2p 3/2 and Cu 2p 1/2.

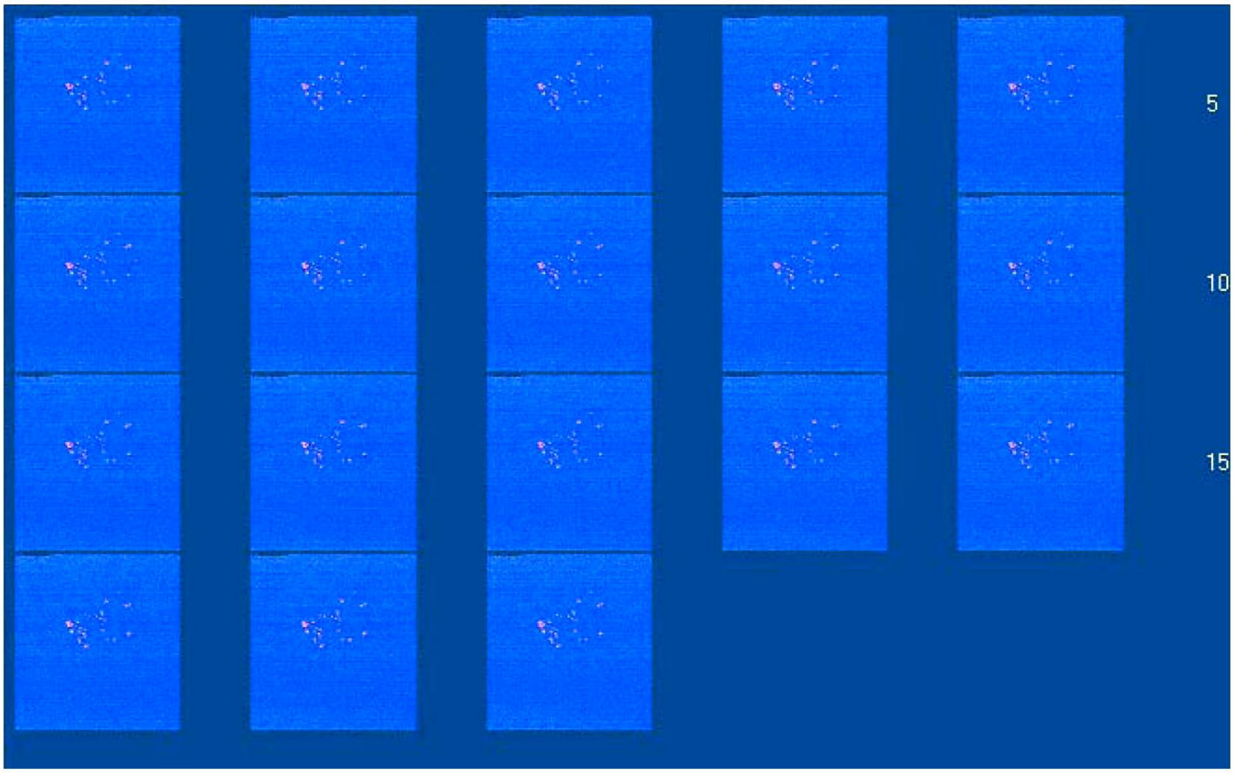

Figure S2│Illuminated LED under blue background. Continuous images of luminous LED with a delay time of 1 s under blue background.

<u>Video (separately attached)</u>:

video1.avi

Video 1│Illumination video. The video of luminous heterojunction LED under blue background, showing the continuous emission of orange-red light.
